# Decellularized Human Stromal Lenticules Combine with Corneal Epithelial-Like Cells: A New Resource for Corneal Tissue Engineering

**DOI:** 10.1155/2019/4252514

**Published:** 2019-12-07

**Authors:** Shuai Qin, Shuai Zheng, Bing Qi, Rui Guo, Guanghui Hou

**Affiliations:** ^1^Department of Ophthalmology, Zhuhai Hospital Affiliated with Jinan University, Zhuhai People's Hospital, Zhuhai, 519000 Guangdong, China; ^2^Department of Spinal Surgery, Nanfang Hospital, Southern Medical University, Guangzhou, 515000 Guangdong, China

## Abstract

The lack of donor corneal tissue or the immunological rejection remains a challenge for individuals with limbal stem cell deficiency (LSCD) who are treated with keratoplasty. Numerous lenticules which were extracted by small incision lenticule extraction (SMILE) appear to be useful materials for keratoplasty. In order to reduce the incidence of allograft rejection, lenticules would be decellularized. Lenticules which were treated with liquid nitrogen and nucleases had no cellular and nuclear materials remained. Human induced pluripotent stem cells (iPSCs) can be generated from the patient who requires keratoplasty, offering an autologous alternative and eliminating the risk of graft rejection. We found that BMP-4, RA, N-2 supplement, hEGF, B27, decellularized human stromal lenticules, conditioned medium, or induction medium promoted the differentiation of human iPSCs with high purity. The results showed that human iPSCs cultured for 4 days in differentiation medium A, 14 days in condition medium, and 1 week in induction medium on decellularized human stromal lenticules developed markedly higher expression of the markers P63, CK3, and CK12 than did those in the other methods. The level of gene expression of the epithelial and pluripotency markers and analysis by scanning electron microscopy and immunohistochemistry also showed successful differentiation. After inducing differentiation in vitro, corneal epithelial-like cells were induced. In the study, we investigated the possibility of a new resource for corneal tissue engineering.

## 1. Introduction

The stratified squamous epithelial cell layer covers the corneal surface, and the maintenance of the healthy corneal epithelium is physiologically achieved by limbal stem cells (LSC) [1]. The cornea is directly exposed to the environment and serious injuries due to various infectious and noninfectious disorders, such as ocular cicatricial pemphigoid, chemical and thermal burns, congenital aniridia and other collagen vascular disease, Stevens-Johnson syndrome, sulfur mustard gas poisoning, chronic inflammation, microbial infections, extended contact lens use, and immune disorders, as well as refractive surgeries [2–10]. These serious conditions must be managed by immediate transplantation to preserve the anatomic integrity of the cornea and prevent complications such as subsequent permanent vision loss and endophthalmitis [10]. However, management of deep corneal defects, especially autoimmune disease, remains a challenge for keratoplasty [11].

Due to the lack of corneal donor tissue or the relatively low 3–5-year graft survival rate, renewable and standardized sources are needed. Human iPSCs can be generated from the patient who requires treatment, offering an autologous alternative and eliminating the risk of graft rejection compared to either autologous or allogeneic limbal epithelial stem cells [12, 13] or expanded ex vivo limbal stem cells [14]. iPSCs are known to have the potential to differentiate into any cell type, and they share similar attributes in terms of morphology, proliferation, differentiation capacity, and genomic and epigenetic states [15, 16]. With numerous patients undergoing SMILE, the extracted lenticules could be used for other treatments [17, 18], such as keratoconus treatment and corrections of hyperopia [19–21], and this method seems to be clinically safe and effective. Decellularized human stromal lenticules provide a powerful three-dimensional (3D) model system, and they display spiralling cell migration patterns in vitro, which are similar to the centripetal movements seen on the corneal surface.

In the present study, we have successfully induced the differentiation of human iPSCs into corneal epithelial-like cells that exhibit a partial retention of the parent cell epigenetic signatures, which are more pronounced in early-passage cells but persist in late passage [22–24]. The application of RA, BMP-4, and small molecule signalling to human iPSCs efficiently mediated epithelial differentiation in conjunction with BMP signalling. Finally, we used this method to generate relatively pure corneal epithelial-like cells, which formed coherent stratified epithelial sheets on decellularized human stromal lenticules, thereby representing a new source for corneal tissue engineering.

## 2. Materials and Methods

This study was approved by the institutional review board of Zhuhai Hospital Affiliated with Jinan University, Zhuhai People's Hospital, Guangdong Province, China, and the research followed the tenets of the Declaration of Helsinki.

### 2.1. Cell Culture

Human iPSCs were prepared from human urothelial cells that were provided by the Key Laboratory of Reproductive Medicine, First Affiliated Hospital of Sun Yat-sen University, within 24 hours of collection. The CytoTune™-iPS 2.0 Sendai Reprogramming Kit (Thermo Fisher Scientific, Life Technologies, Carlsbad, CA, http://www.thermofisher.com) loaded with the Yamanaka 4 factors Oct3/4, Sox2, c-MYC, and Klf4 was used to reprogram the epithelial cells [25]. Human iPSCs were maintained in Essential 8™ Medium, and the medium was changed every 1-2 days. Human iPSCs were harvested using 50 mL of 0.5 mM EDTA in DPBS. Then, the cells were washed with DPBS, mechanically dissociated into smaller clumps, and seeded onto matrix-coated 6-well plates.

**Figure 1:**
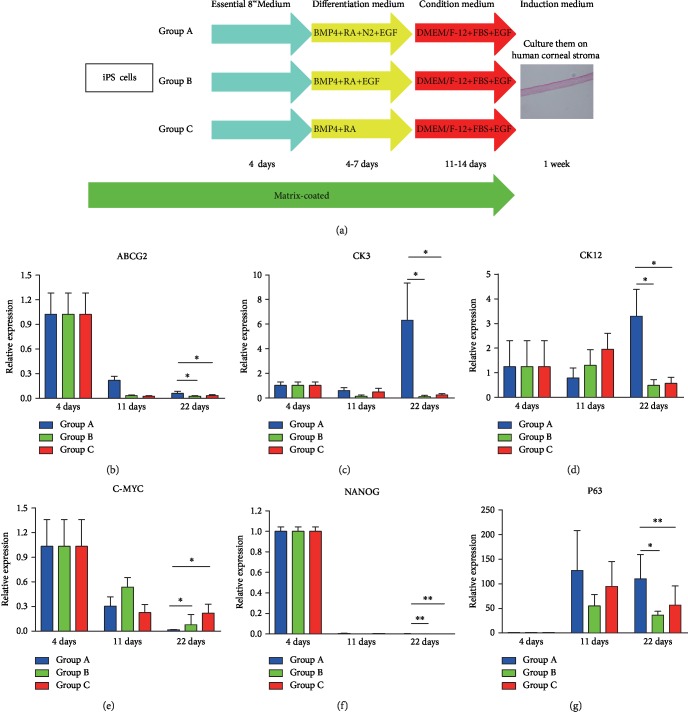
The induction process is shown (a). Quantitative real-time PCR for the amplification of epithelial cell markers and pluripotency markers in induced pluripotent stem cell cultures in group A, group B, and group C (4 days, 11 days, and 22 days, b–g). All three groups exhibit increased expression of P63 but decreased expression of ABCG2, c-MYC, and NANOG. The relative gene expression of CK3, CK12, ABCG2, and c-MYC was significantly different among the three groups (paired *t*-test; *p* < 0.05, ^∗^*p* < 0.05, and ^∗∗^*p* > 0.05).

The human iPSCs were maintained in Essential 8™ Medium (Thermo Fisher Scientific) and 1% penicillin-streptomycin (Gibco). Differentiation medium A consisted of Dulbecco's modified Eagle's medium : Nutrient Mixture F-12 (DMEM/F-12) (Gibco) and N-2 Supplement (100x) (Thermo Fisher Scientific) to achieve 1 *μ*M all-trans retinoic acid (Sigma), 25 ng/mL BMP-4 (R&D Systems, Minneapolis, MN), 10 ng/mL recombinant human epidermal growth factor (hEGF) (Thermo Fisher Scientific), and 50 U/mL penicillin-streptomycin (Thermo Fisher Scientific) diluted to a final working concentration. Differentiation medium B consisted of Defined Keratinocyte-SFM (Thermo Fisher Scientific), to achieve 1 *μ*M all-trans retinoic acid, 25 ng/mL BMP-4, 10 ng/mL recombinant human epidermal growth factor, and 50 U/mL penicillin-streptomycin diluted to a final working concentration. Differentiation medium C consisted of Defined Keratinocyte-SFM diluted to achieve 1 *μ*M all-trans retinoic acid and 25 ng/mL BMP-4 diluted to a final working concentration [25, 26]. The condition medium consisted of DMEM/F-12, 10% FBS, 10 ng/mL hEGF, and an antibiotic-antimycotic mixture (Thermo Fisher Scientific) [26, 27]. The induction medium consisted of DMEM/F-12, 10% FBS, 10 ng/mL hEGF, B27 supplement, and other components. All media were filter sterilized using a 0.22 m filter (Millipore, Billerica, MA, http://www.millipore.com) and stored at 4°C. The induction process is shown in Figure 1(a).

The human iPSCs were randomly divided into group A, group B, and group C. In group A, cells were maintained in Essential 8™ Medium for 4 days, aspirated off Essential 8™ Medium from the dish, and added to differentiation medium A. The medium was replaced after 48 h of induction. After the second 48 h of induction, the cells were cultured with the condition medium for 14 days. After that, the cells were cultured on decellularized human stromal lenticules with the induction medium for 7 days. In group B, cells were maintained in Essential 8™ Medium for 4 days, aspirated off Essential 8™ Medium from the dish, and added to differentiation medium B. The medium was replaced after 48 h of induction. After the second 48 h of induction, the cells were cultured with the condition medium for 14 days. After that, the cells were cultured on decellularized human stromal lenticules with the induction medium for 7 days. In Group C, cells were maintained in Essential 8™ Medium for 4 days, aspirated off Essential 8™ Medium from the dish, and added differentiation medium C. The medium was replaced after 48 h of induction. After the second 48 h of induction, the cells were cultured with the condition medium for 14 days. After that, the cells were cultured on decellularized human stromal lenticules with the induction medium for 7 days.

On day 22 of differentiation, the cells were collected into a 15 mL conical tube. The cell supernatant was discarded after centrifugation at 200 × *g* for 6 min. The pellet was resuspended with the induction medium, and 200 *μ*L of cell suspension was added into the 96-well plates which contained the decellularized human stromal lenticules (8 × 10^4^ cells per well). The induction medium was changed every day. After 7 days on the decellularized human stromal lenticules, the cells were washed with 1× PBS, added with Trypsin-EDTA (0.25%), and incubated in the incubator until cells start detaching; then, the cell suspension was collected into a 15 mL conical tube. The number of cells on 10 pieces of lenticules was about 1.3 × 10^6^.

### 2.2. Decellularized Human Stromal Lenticules

With written informed consent, human stromal lenticules were obtained after femtosecond laser refractive surgery (approved research project IRB review board protocol Pro2018001). Then, the tissues were thoroughly washed in PBS three times, digested with 0.025% Trypsin-EDTA for 60 min at 37°C, and then placed in -196°C liquid nitrogen for 15 min. Next, the lenticules were placed in a 37°C water bath quickly after thawing, and the freeze/thaw process was repeated three times. The tissues were put in DNA and RNA enzyme (667 mu kat/L) digestion for 1 h, washed three times, and moved to a freeze-drying machine with vacuum drying for 12 h after being prechilled in the freezer (-80°C). Finally, the tissues were sealed in sterile plastic bags (Co60 disinfected) and saved for later use. A freeze-dried human stromal lenticules look similar to a white loose flake, but when they are placed in DMEM for 4 h, they become translucent and exhibits toughness, thickness, and strength similar to those of a normal cornea.

### 2.3. Quantitative RT-PCR

The corneal epithelial cells can be distinguished by the expression of CK3, CK12, ABCG2, c-MYC, P63, and Nanog [28–32]. LightCycler capillary tubes (Roche Diagnostics, Basel, Switzerland, http://www.roche-applied-science.com) were placed in cooled centrifugation tubes (Roche) to confirm the successful infection by real-time PCR, which was performed using SYBR® Premix Ex Taq™ II (Takara, Japan). Each capillary tube was filled with 2 *μ*L of cDNA, 10 *μ*L of SYBR® Premix Ex Taq™ II (Takara), 0.8 *μ*L each of the specific 10 *μ*M forward and reverse primers (Thermo Fisher Scientific), and 6.4 *μ*L of sterile water (Takara). The real-time PCR reaction was performed using the LightCycler with the following conditions: 95°C for 30 s, followed by 40 cycles of 95°C for 5 s and 60°C for 30 s, primer-specific annealing/extension temperature, 95°C for 5 seconds, and 60°C for 1 minute, with a single data acquisition step. The cycle threshold for each transcript was determined using the LC480 PCR instrument (LightCycler® 480 II) using three-stage program parameters provided by the manufacturer (Roche), and the Relative Quantification Software (Roche) was used to analyse the data. GADPH and the undifferentiated human iPSCs were examined for each gene investigated. Cycle threshold (CT) values were obtained from the logarithmic amplification phase, and the relative quantification of each gene was calculated using the 2-ΔΔCt method [33]. All assays included 3 biological replicates for each time point of differentiation. The primers used for real-time PCR are shown in Table 1.

### 2.4. Immunocytochemistry Staining

The cells were washed in phosphate-buffered saline and fixed with 4% p-formaldehyde for 20 min and permeabilized with 0.3% Triton X-100 (Sigma-Aldrich) for 5 min, followed by three washes with PBS. Next, the cells were blocked in goat serum (Sigma-Aldrich) for 20 min and incubated with the primary antibody (CK3/12, P63) diluted in blocking solution (dilution 1 : 100 or dilution 1 : 50) overnight at 4°C. The next day, the primary antibody was removed from the well, and the well was incubated with PBS three times for 5 minutes at room temperature. Then, the cells were stained with the appropriate fluorophore-conjugated secondary antibody diluted in blocking solution for 1 h in the dark. Finally, the cells were washed with PBS and counterstained with DAPI, which contained mounting medium. The stained cells were observed using fluorescent microscopy (Zeiss, Germany) and edited using ZEN imaging software. The fluorescence intensities were quantified using ImageJ software (available at http://rsb.info.nih.gov/ij).

### 2.5. Scanning Electron Microscopy

Circular cover glasses were placed in the culture wells for the culturing of differentiating human iPSCs. The differentiating human iPSCs were established on the coverslips, which were removed from the culture wells on day 22 of culture and fixed in 2.5% glutaraldehyde in Sorenson's phosphate buffer overnight at 4°C. The cover glasses with the fixed cultures were then washed three times in PBS for 15 minutes, dehydrated at room temperature in sterile water for 30 minutes each in 25, 50, and 75% ethanol (BDH), and finally stored in 100% ethanol at 4°C before processing. The cultures on the cover glass were then further dehydrated with carbon dioxide in a Samdri 780 Critical Point Dryer. The cover glasses were mounted on an aluminium stub using Acheson Silver Electrodag (Agar Scientific), and the cultures on the coverslips were subsequently coated with 15 nm gold using a polaron scanning electron microscopy coating unit (Empdirect, Houston, TX). The specimens were examined using a StereoScan 240 SE microscope, and photographs were taken (Leica).

### 2.6. Western Blots

Cellular proteins were harvested using RIPA buffer (Santa Cruz Biotechnology) and protease inhibitors (Roche, Paris, France). The extracted proteins were subjected to SDS polyacrylamide gel electrophoresis, followed by immunoblotting. The antibodies used were rabbit anti-P63 (1 : 1000) (Abcam), mouse anti-CK3+12 (1 : 1000) (Abcam), and rabbit anti-c-MYC (1 : 1000) (Abcam). After blocking with 5% milk in PBS, the membranes were probed with primary antibodies overnight and then stained with horseradish peroxidase-conjugated antibodies for 1 hour. The proteins were visualized by enhanced chemiluminescence (Pierce), protein loading was verified by probing against GAPDH, and expression was quantified by densitometric analysis with Image Master VDS-CL using TINA 2.0 software (ray tests).

### 2.7. Histological and Immunofluorescent Analysis

Human iPSCs were grown on the surface of decellularized human stromal lenticules. After an entire cell sheet was formed by the differentiated human iPSCs, the cells were fixed in 4% paraformaldehyde. The grown samples were dehydrated and embedded in paraffin, after which 3 mm thick longitudinal sections were obtained for staining with haematoxylin and eosin (H&E), according to standard laboratory protocols. Immunofluorescent staining was performed as previously described. The following primary antibodies were used: rabbit anti-K3 (1 : 50) and rabbit anti CK12 (1 : 50). The fluorescent Alexa 594-conjugated secondary antibody was used for CK3 and CK12. Immunofluorescent images were acquired using a model TCS SP2 confocal laser-scanning microscope (Leica Microsystems).

### 2.8. Statistical Analysis

The statistical analysis was performed using two-way ANOVA (followed by a paired *t*-test as the post hoc test). The results are expressed as the mean ± SD; results were considered significant at *p* < 0.05.

## 3. Results and Discussion

### 3.1. Results

#### 3.1.1. Human iPSC Differentiation Confirmed by Downregulation of Pluripotency Markers and the Upregulation of the Corneal Epithelial Marker

To investigate the effect of the media on early-stage differentiation, the expression of several genes was studied using quantitative PCR (qPCR). We investigated the expression of key genes, including P63, CK3, CK12, ABCG2, c-MYC, and Nanog. We analysed the expression of these genes in the human iPSCs in every procedure during the differentiation process. The differentiation process was evaluated over a 22-day period, during which the expression of the undifferentiated stem cell markers ABCG2, Nanog, and c-MYC decreased (Figures 1(b), 1(e), and 1(f)), coupled with an increase in the expression of several genes for corneal epithelial markers, CK3, CK12, and P63 (Figures 1(c), 1(d), and 1(g)). Therefore, the three groups had undergone differentiation compared to human iPSCs. The downregulation of ABCG2 and c-MYC and the upregulation of CK3 and CK12 in group A were significantly more pronounced compared to other groups (2-way ANOVA; ^∗^*p* < 0.05, ^∗∗^*p* > 0.05), suggesting a higher extent of differentiation in group A.

### 3.2. Morphological Changes in the Differentiated Human iPSCs

**Figure 2:**
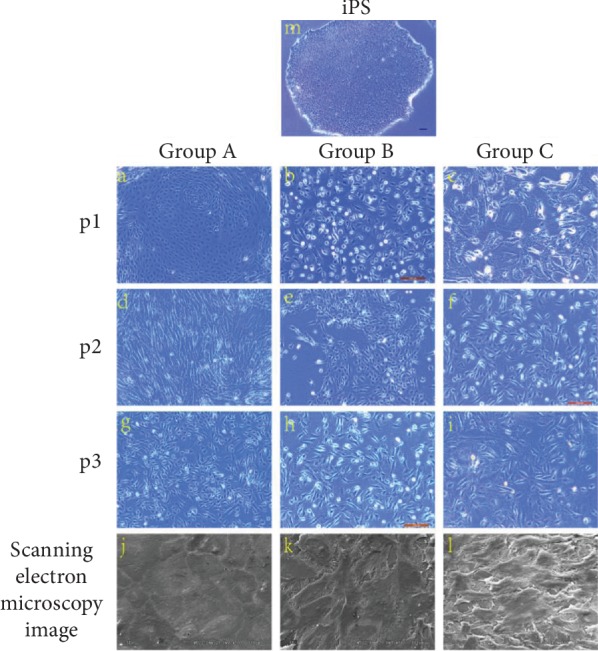
Morphological analysis was performed for iPSCs and corneal epithelial-like cells in three groups for P1, P2, and P3 after the cells were differentiated in culture. In all the three groups, the iPSCs flattened and changed from a round sphere-like morphology to the cobblestone appearance typical of HCECs (a–i). Scale bars = 100 *μ*m. Scanning electron microscopy image from 22 days of differentiation in group A, group B, and group C. Note the numerous microcilia. Magnification, ×500 (j–l). The photograph is taken of human iPSCs (m).

Morphological analysis was performed on human iPSCs and corneal epithelial-like cells in the three groups (P1, P2, and P3) after the cells were differentiated in the culture. In the three groups, the iPSCs began to flatten and changed from a round sphere-like morphology to the cobblestone appearance typical of HCECs (Figures 2(a)–2(i) and 2(m)). The morphological changes in both the iPSCs and P1 cell colonies during the differentiation time period studied were very similar (Figures 2(a) and 2(m)). Scanning electron microscopy was used to observe the iPSCs that had differentiated for 22 days using differentiation medium and condition medium. The most significant similarity among the three groups, which was observed using scanning electron microscopy, was the presence of microcilia similar to those on epithelial cells that have multiple microcilia (Figures 2(j), 2(k), and 2(l)). Importantly, the undifferentiated iPSCs did not exhibit microcilia (data not shown).

### 3.3. Immunological and Proteomic Changes in the Differentiated Human iPSCs

**Figure 3:**
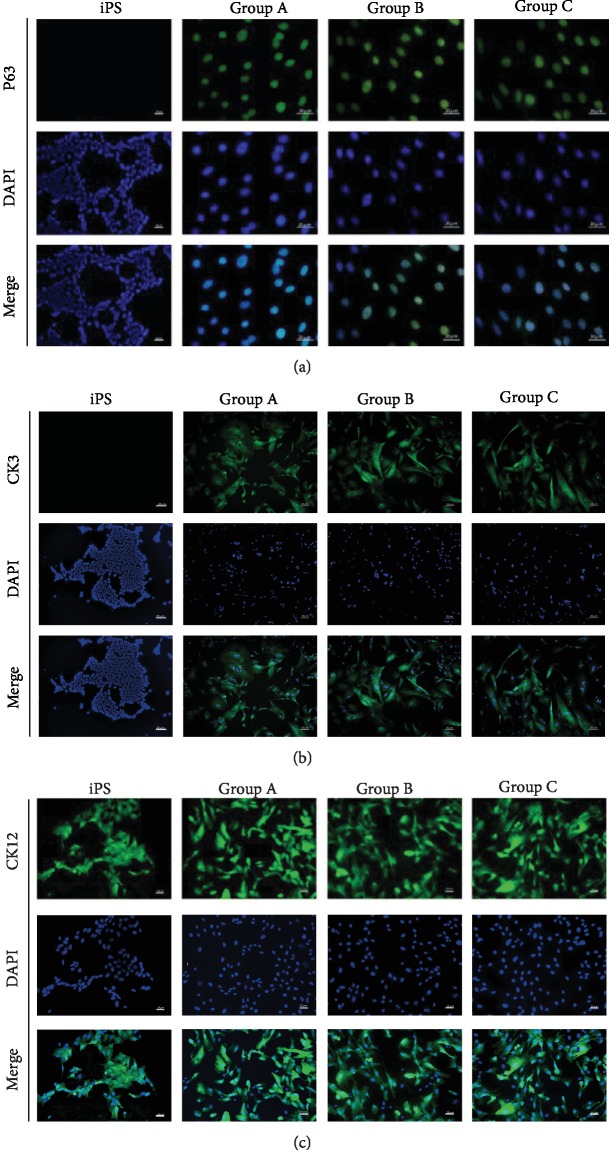
After 22 days in the differentiation cultures, cells in all the three groups expressed the corneal epithelial progenitor marker p63 (a). After 22 days in the differentiation culture, cells in all the three groups expressed the corneal epithelial markers CK3 and CK12 (b, c). The scale bars represent 20 *μ*m.

**Figure 4:**
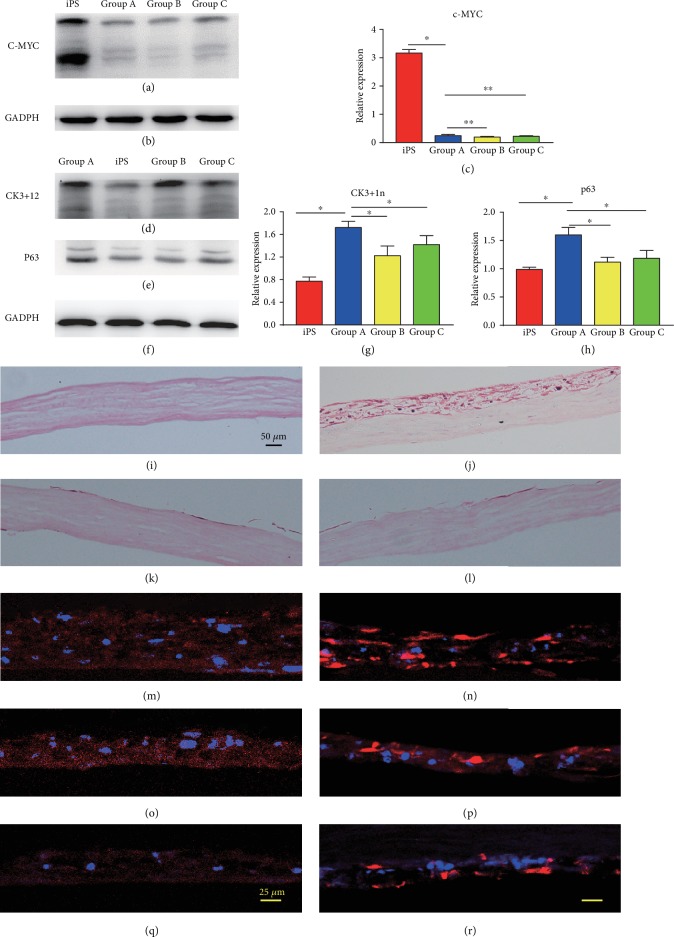
The expression levels of c-MYC (a–c) were significantly higher in the iPSC group than in the other groups. In contrast, the results of western blotting showed that the protein levels of CK3+12 (d, f, g) were higher in group A than in the other groups, as well as P63 (e, f, h). Decellularized human corneal stroma had no cells reserved (i). After 32 days in the differentiation culture, grafts grown on decellularized corneal stroma exhibited cell outgrowths and formed a stratified epithelial layer within 14 days of cultivation in group A (j). Few cells remained on the decellularized corneal stroma in group B and group C (k, l). The expression of the markers of terminally differentiated corneal epithelial cells, K3 (m) and K12 (n). Group B and group C also expressed the markers K3 (o, q) and CK12 (p, r), but the expression was lower than that in group A. Magnification: ×100 (i–l) and ×200 (m–r). The scale bars represent 50 *μ*m (i–l). The scale bars represent 25 *μ*m (m–r).

To verify whether our human iPSCs differentiated into corneal epithelial-like cells, we performed immunocytochemistry to detect corneal epithelial markers. After 22 days in the differentiation culture, the human iPSCs expressed the corneal epithelial progenitor marker p63 (Figure 3(a)), the corneal epithelial progenitor marker CK3 (Figure 3(b)), and the corneal epithelial marker CK12 (Figure 3(c)), whereas the expression of CK3, CK12, and P63 was stable and high over the 22 days. Conversely, cells that were differentiated under spontaneous conditions did not express any of the abovementioned corneal epithelial markers (data not shown). The results of western blotting showed that the protein levels of c-MYC (Figures 4(a) and 4(b)) were higher in the iPSC group than in the other groups. In contrast, the expression levels of CK3+12(Figures 4(d) and 4(f)) and P63 (Figures 4(e) and 4(f)) were significantly higher in group A than in the other groups.

### 3.4. Morphological and Immunological Changes in Tissues

We first tried to induce iPSCs towards corneal epithelial-like cells by culturing them on decellularized human stromal lenticules after 22 days of differentiation. At 29 days, cells and tissues were harvested and subjected to H&E and immunofluorescent staining. The decellularized human corneal stroma had no cells remaining (Figure 4(i)). After 29 days in differentiation culture, grafts grown on decellularized corneal stroma exhibited cell outgrowths and formed a stratified epithelial layer within 7 days of cultivation in group A (Figure 4(j)). Few cells remained on the decellularized corneal stroma in group B and group C (Figures 4(k) and 4(l)). Among the tested groups, group A enhanced the differentiation of human iPSCs into corneal epithelial-like cells, as illustrated by the increased expression of the marker of terminally differentiated corneal epithelial cells, CK3 (Figure 4(m)), and the marker of epithelial cells, CK12 (Figure 4(n)). Other groups also exhibited an increase in the expression of the markers K3 (Figures 4(o) and 4(q)) and CK12 (Figures 4(p) and 4(r)), but the increase was lower than that of group A. The N2-supplemented medium promoted greater differentiation than the other media.

## 4. Discussion

Limbal epithelial stem cell (LESC) deficiency (LSCD) leads to corneal abnormalities that result in compromised vision and blindness. Autologous transplantation is not feasible for bilateral LSCD cases, allogeneic transplantation is not feasible for immunological rejection cases, and a reliable banked source of LESC is needed. LSCD can potentially be treated by transplantation of appropriate cells, which should be easily expandable and bankable. The differentiated hiPSCs do not remain immunogenic, and thus, the recipient will not require immunosuppression. Therefore, these cells are a promising option for LSCD treatment.

Nowadays, stem cell research and culture techniques open up a new field of keratoplasty. Cellular therapy presents challenges such as maintaining phenotypic stability, cell viability, and certain regulatory issues specific to the application of living, allogeneic cells to the eye [34]. Embryonic stem cells (ESCs) or human iPSCs seeded on corneas have failed to stratify [35, 36]. In the experiment, we found that iPSCs in differentiation medium A had less apoptosis and better state than the other two groups and proved that in the process of differentiation of iPSCs, N2 could reduce the incidence of apoptosis and keep cells in a good condition. What is more, human iPSCs induced towards corneal epithelial-like cells seeded on a decellularized cornea and differentiated with bone morphogenetic protein; N2 and all-trans retinoic acid produced the stratified epithelium expressing epidermal markers, providing a new source for corneal tissue engineering.

The purpose of this study was to investigate a new resource for corneal tissue engineering, by mimicking the corneal epithelia and stroma within the in vitro culture system. Differentiated human iPSC grafts grown on decellularized corneal stroma exhibited cell outgrowths and formed a stratified epithelial layer, indicating that these cells are a promising source for LSCD treatment. This progression, in particular, to the immunological rejection cases for which allogeneic transplantation is not feasible, and a reliable source of tissue engineering cornea is needed. Decellularized human stromal lenticules could provide a suitable scaffold for the survival and proliferation of corneal epithelial-like cells, which formed a continuous epithelium with the expression of characteristic epidermal markers. Furthermore, in vitro cell culture revealed that differentiated iPSC grafts grown on decellularized human stromal lenticules exhibited cell outgrowths and formed a stratified epithelial layer, indicating that these cells are a promising source for keratoplasty. The differentiated human iPSCs and decellularized human stromal lenticules do not remain immunogenic and will not require immunosuppression. Therefore, decellularized human stromal lenticules combine with corneal epithelial-like cells as the source, with translational potential for keratoplasty.

The transcription factors Nanog and c-MYC are critically involved in the self-renewal of undifferentiated stem cells, whereas corneal epithelium cells specifically express P63, CK3, and CK12; iPSCs express Nanog and c-MYC, but not CK3 and CK12. These markers are therefore critical for identifying the differentiated iPSCs. The expression levels of corneal epithelial markers CK3 and CK12 were consistently higher in group A than in other groups for 22 days. These data were further verified by immunostaining using sections of decellularized human stromal lenticules with 29-day-old differentiated iPSCs. Importantly, group A developed high expression of the markers CK3 and CK12. Immunostaining for the conjunctival marker K14 revealed its insignificant expression in all groups (data not shown). These corneal epithelial-like cells (CK3+/CK12+/P63+/Nanog-/ABCG2-/c-MYC-) retained the ability to further differentiate into corneal epithelial cells upon treatment with N2, hEGF, RA, BMP4, decellularized human corneal stroma, and so on. These results also demonstrate that the iPSCs generated by these treatments are capable of further differentiation upon receiving the proper cues. The model of iPSC differentiation that we have developed in this article may aid in our understanding of the early events of epithelial lineage specification and the eventual potential application of epithelial-like cells differentiated from iPSCs.

Indeed, here, we show that differentiated human iPSCs in the loss of pluripotency and the differentiation into epithelial-like cells. We found that RA and BMP-4, coupled with conditioned medium and decellularized human stromal lenticules, allowed human iPSCs to differentiate in a manner that recapitulated corneal epithelial lineage development with high purity. Scanning electron microscopy revealed that some differences existed among the groups. It is known that the ciliary localizes several cell signaling molecules and receptors and helps in sensing extracellular signals to regulate various cellular functions [37–39]. In future studies, in vivo animal models are warranted.

## 5. Conclusions

Human iPSCs differentiated into corneal epithelial-like cells on decellularized human stromal lenticules, which was mimicked within the in vitro culture system. Such iPSCs and decellularized human stromal lenticules could become a new expandable and bankable source for transplantation. Our study not only contributes important new discoveries for the basic research field of corneal epithelial development but also introduces a strategy to develop corneal epithelial cells that have great potential in clinical regenerative medicine to treat damaged corneal epithelia.

## Figures and Tables

**Table 1:** Real-time PCR primers.

Gene	Forward primer (5′–3′)	Reverse primer (5′–3′)
CK3	TTAAGGACCCTCTACGACGC	AATGATGCTGTCCAGGTCCA
CK12	TGGAGATTGAGACCTACCGC	ACCATTCACCATCTCCTGCA
P63	TCCATGGATGATCTGGCAAGT	GCCCTTCCAGATCGCATGT
Nanog	AGAAGGCCTCAGCACCTAC	GGCCTGATTGTTCCAGGATT
c-MYC	GCGTCCTGGGAAGGGAGATCCGGAGC	TTGAGGGGCATCGTCGCGGGAGGCTG
ABCG2	AACCTGGTCTCAACGCCATC	GTCGCGGTGCTCCATTTATC
GADPH	GTGGACCTGACCTGCCGTCT	GGAGGAGTGGGTGTCGCTGT

## Data Availability

The data used to support the findings of this study are available from the corresponding author upon request.
